# Psychological consequences of war in Ukraine: assessing changes in mental health among Ukrainian parents

**DOI:** 10.1017/S0033291723000818

**Published:** 2023-11

**Authors:** Philip Hyland, Frédérique Vallières, Mark Shevlin, Thanos Karatzias, Menachem Ben-Ezra, Eoin McElroy, Maria Louison Vang, Boris Lorberg, Dmytro Martsenkovskyi

**Affiliations:** 1 Department of Psychology, Maynooth University, Kildare, Ireland; 2 Trinity Centre for Global Health, Trinity College, University of Dublin, Dublin, Ireland; 3 School of Psychology, Ulster University, Derry, UK; 4 School of Health & Social Care, Edinburgh Napier University, Edinburgh, UK; 5 School of Social Work, Ariel University, Ariel, Israel; 6 Centre for Psychotraumatology, University of Southern Denmark, Odense, Denmark; 7 Department of Psychiatry, UMass Chan Medical School, Worcester, Massachusetts, USA; 8 Department of Psychiatry and Narcology, Bogomolets National Medical University, Kyiv, Ukraine; 9 SI ‘Institute of Psychiatry, Forensic Psychiatric Examination and Drug Monitoring of Ministry of Health of Ukraine’, Kyiv, Ukraine

Russia's ongoing war on Ukraine has profoundly changed the lives of millions of people (United Nations, 2023), and the mental health community has warned of potential long-term negative mental health consequences (Morganstein, Bromet, & Shigemura, 2022). The adverse mental health effects of war are well-established (Jain et al., 2022), and there is evidence that Russia's 2014 invasion of the south and east of Ukraine (Shevlin et al., 2018) and the full-scale invasion in 2022 (Karatzias et al., 2023) has led to serious psychological harm among the population. In this study, we report on changes in anxiety, depression, loneliness, and hazardous drinking following the 24 February 2022 invasion, and the correlates of changes in these mental health problems.

Data were collected online from a nationwide sample of parents living in Ukraine (*N* = 2004) between 15th July and 5th September 2022. Ethical approval was provided by the SI Institute of Psychiatry, Forensic Psychiatric Examination, and Drug Monitoring which is part of the Ministry of Health in Ukraine. Participants were recruited from oblasts in every region of Ukraine (28% from northern regions, 25% from western regions, 24% from southern regions, 18% from central regions, and 5% from eastern regions), 57.1% were female, the mean age was 37.7 years (s.d. = 8.19), the majority were in a committed relationship (78.0%), university educated (62.7%), and in full- or part-time employment (59.4%).

Participants completed modified versions of the Generalized Anxiety Disorder 7-item Scale (GAD-7: Spitzer, Kroenke, Williams, & Löwe, 2006), Patient Health Questionnaire-9 (PHQ-9: Kroenke, Spitzer, & Williams, 2001), Three-Item Loneliness Scale (TILS: Hughes, Waite, Hawkley, & Cacioppo, 2004), and the 10-item Alcohol Use Disorders Identification Test (AUDIT: Babor, La Fuente, Saunders, & Grant, 1992). Instruction were modified to read: ‘In this section we will ask you about how your mental health has changed since the war on Ukraine began on February 24th, 2022. Please read the following items and indicate if these have happened more frequently since the war began’. The Likert response scales were changed to include five options: ‘No change or less’ (0), ‘A little more often’ (1), ‘Slightly more often’ (2), ‘A lot more often’ (3), and ‘A great deal more often’ (4). Mean change scores from 0 to 4 were computed for each variable. These measures were translated from English into Ukrainian and then back-translated to ensure accuracy by a team of experts fluent in both languages. Internal reliability in the current sample was excellent: GAD-7 (*α* = 0.93), PHQ-9 (*α* = 0.90), TILS (*α* = 0.86), and AUDIT (*α* = 0.92). Exposure to 34 war-related events was also assessed. The mean number of exposures was 9.07 (s.d. = 4.35) and a quintile variable was used for analytic purposes.

One-sample *t* tests indicated that mean changes in symptoms of anxiety (*M* = 1.62, s.d. = 0.98; *t*(2003) = 73.96, *p* < 0.001; *d* = 1.65), depression (*M* = 1.34, s.d. = 0.87; *t*(2003) = 69.15, *p* < 0.001; *d* = 1.54), loneliness (*M* = 1.02, s.d. = 1.03; *t*(2003) = 73.96, *p* < 0.001; *d* = 0.99), and hazardous alcohol use (*M* = 0.23, s.d. = 0.51; *t*(2003) = 20.35, *p* < 0.001; *d* = 0.46) were significantly different from zero with effect sizes in the ‘moderate’ or ‘large’ range. Additionally, 71.9% of participants reported increased symptoms of anxiety, 62.1% reported increased symptoms of depression, 46.8% reported increased feelings of loneliness, and 7.8% reported increased symptoms of hazardous alcohol use. Path analysis was used to identify factors associated with changes in each mental health problem and the results are presented in Table 1. Notably, changes in anxiety, depression, loneliness, and hazardous alcohol use were all related to exposure to a higher number of war-related stressors, and each followed a dose–response pattern.

**Table 1.** Standardized regression coefficients (*β*) for change in mental health symptoms

	ΔAnxiety	ΔDepression	ΔLoneliness	ΔAlcohol use
Male sex	−0.31***	−0.26***	−0.09***	0.13***
Age	−0.01	−0.00	−0.06*	−0.08***
Number of children	−0.01	−0.01	−0.05*	−0.01
Child with EBP	0.09***	0.09***	0.09***	0.06*
Single	0.03	0.04	0.05*	0.00
Not living with partner	0.00	0.03	0.05*	0.02
Separated or divorced	−0.03	−0.01	0.09*	0.02
Widowed	−0.01	0.02	0.02	0.01
Completed secondary school	0.01	−0.01	0.01	0.04
Completed vocational school	−0.01	−0.03	−0.01	0.01
Part-time employed	0.01	−0.01	−0.00	0.02
Unemployed due to the war	−0.00	0.03	0.08**	−0.01
Unemployed not due to the war	0.03	0.02	0.04	−0.02
Student	−0.03	−0.02	−0.00	−0.01
Retired	−0.03	−0.04*	−0.01	−0.01
Not working due to disability	−0.00	0.00	0.03	−0.02
Emergency worker – self	0.00	−0.01	−0.03	−0.01
Emergency worker – relative	0.01	0.02	−0.01	−0.01
Western Ukraine	0.06*	0.09***	0.06*	0.03
Central Ukraine	0.07**	0.10***	0.03	0.04
Eastern Ukraine	0.03	0.04	0.01	0.03
Southern Ukraine	0.05*	0.09***	−0.00	0.01
Living in an urban area	0.03	0.01	0.00	0.04
Living in an apartment	0.00	−0.00	0.02	0.01
In emergency accommodation	0.02	0.03	0.02	0.03
War experiences 2nd quintile	0.12***	0.12***	0.09***	0.03
War experiences 3rd quintile	0.16***	0.17***	0.11***	0.08**
War experiences 4th quintile	0.27***	0.26***	0.17***	0.11***
War experiences 5th quintile	0.37***	0.35***	0.26***	0.14***
*R*^2^	0.205***	0.173***	0.102***	0.047***

Δ, change; EBP, emotional or behavioural problem; *R*^2^, percentage of variance explained.

Statistical significance: *p < 0.05, **p < 0.01, ***p < 0.001.

These results indicate that most participants experienced worsening mental health following Russia's attack in February 2022, with the greatest increases being evident for anxiety, depression, and loneliness. More modest changes were reported for hazardous drinking, but this may have been influenced by the temporary ban on the sale of alcohol due to enactment of martial law. These findings contribute to a growing evidence base that the Russian war on Ukraine has led to increased mental health problems in the population (Karatzias et al., 2023). Continued monitoring of the population will be needed to determine if these changes reflect a normal and temporary response to an extreme change in the environment, or if they mark the beginning of longer-lasting mental health problems.

Parents caring for a child with emotional or behavioural problems reported greater increases in all mental health problems. Caring for a child with emotional and behavioural problems is known to be associated with elevated levels of stress (Cousino & Hazen, 2013), and in the context of an ongoing war this appears to be associated with worsening mental health. Increases in anxiety, depression, and loneliness were higher for women, while increases in hazardous drinking were higher for men, highlighting potentially important differences in how the sexes are responding to the war. Younger adults also reported greater changes in feelings of loneliness and hazardous drinking, while those who were retired reported less change in depression. These findings suggest that the disruption to normal life brought about by the war may be disproportionately affecting younger people. We also found regional differences with those living in the west, centre, and south of Ukraine reporting more pronounced changes in their mental health compared to those living in the north. It is difficult to know why this might be but it is possible that living in the north of the country – which includes the strongly fortified capital city of Kyiv – offered a greater sense of protection and safety.

The non-representative nature of the sample, and the small number of mental health measures employed limits the generalizability of the findings. Nonetheless, we hope these findings prove useful in documenting the mental health effects of Russia's war on Ukraine, and in informing humanitarian mental health responses by highlighting those that are potentially most in need of mental health care.
